# Clinical, Histological, and Scintigraphic Comparative Study of the Use of Mandibular Bone Marrow and Peripheral Blood in Bone Neoformation

**DOI:** 10.1155/2021/4867574

**Published:** 2021-12-31

**Authors:** Paulo José Pasquali, Rodrigo André Dall'Agnol, Lucas Garcia Rezende, Elizabeth Ferreira Martinez

**Affiliations:** ^1^Division of Implantology, Santa Mônica Hospital Center, Erechim, RS, Brazil; ^2^Division of Implantology, Faculdade São Leopoldo Mandic, Campinas, Brazil; ^3^Division of Orthopedics and Traumatology, Saint Hill Hospital, Caçador, SC, Brazil; ^4^Division of Nuclear Medicine, Ernesto Dornelles Hospital, Porto Alegre, RS, Brazil; ^5^Division of Oral Pathology and Cell Biology, São Leopoldo Mandic Research Institute, Campinas, SP, Brazil

## Abstract

**Materials and Methods:**

The study included 16 patients with maxillary atresia. The region was grafted with xenograft blocks associated with the following treatments: G1, the patient's peripheral blood during surgery, and G2, dripping of mandibular bone marrow blood until the xenograft was completely wet. After 7 and 14 days, scintigraphic images of the regions of interest (ROI) were taken to quantify pixels, which indicate osteogenic activity. Additionally, trephined samples obtained at the time of implant placement were stained in H&E, and newly formed bone tissue was quantified. The data were tabulated and statistically analyzed at a significance level of 5%.

**Results:**

Scintigraphic data showed greater osteogenic activity with mandibular bone marrow blood (G2) at all times evaluated (*p* < 0.05). As for the histomorphometric analysis, a greater amount of bone tissue was observed in samples treated with mandibular bone marrow blood (G2) compared to peripheral blood (G1) (*p* < 0.05).

**Conclusions:**

The appositional bone reconstruction technique in the block associated with mandibular bone marrow blood increased bone neoformation and osteogenic activity compared to conventional graft treatment with peripheral blood.

## 1. Introduction

Although the autologous bone graft obtained from the intraoral region is a safe option to restore bone volume, it presents undesirable postsurgical comorbidities [1]. Thus, the use of biomaterials has been widely used as an alternative to autologous grafts [2].

However, bone substitutes have deficient osteogenic and osteoinductive capacities, which can affect the success of the procedure [3]. The lack of cellularity observed in bone substitutes has been arousing interest in tissue engineering research, which aims at associating osteogenic autologous cells with osteoconductive biomaterials. In this context, bone marrow represents a promising source of autologous cells, containing a vast cellular component [4, 5], which combined with the angiogenic potential [6] improves tissue regeneration and graft integration [7].

Autologous bone marrow concentrate has known benefits due to the wide variety of cells, including endothelial progenitor cells, mesenchymal stem cells (MSCs), hematopoietic stem cells (HSCs), and other progenitor cells. In addition to the cellular component, it contains growth factors and chemokines, including platelet-derived growth factor, bone morphogenetic protein, transforming growth factor-beta, vascular endothelial growth factor, interleukin (IL)-8, and IL-1 receptor antagonist [8–11]. In addition, bone marrow aspiration to collect cells is relatively easier than using autologous bone graft from the same donor site, making it a very versatile source [12].

Of the cellular bone marrow components, there has been a greater interest in the use of stem cells for bone regeneration, since in vitro and in vivo studies have shown highly promising results [13, 14]. In addition to originating blood cells, bone marrow may have the potential to induce undifferentiated cells to differentiate into a variety of tissues, including the bone and cartilage [15].

In this context, studies aiming to search for bone donor sites with less comorbidities for bone grafting have been disseminated in recent times, with emphasis on the use of mandibular bone marrow blood. Collecting mandibular bone marrow blood for intraoral bone grafts is easier as it comes from a site that is being manipulated in the transsurgical period [16]. Furthermore, it has stem cells with a high capacity for osteogenic differentiation, being considered an important enhancer in osteogenic grafts [17].

In addition to the cellular potential, it has the capacity to promote vascularization through the use of mandibular bone marrow blood, which, combined with the use of biomaterials with good osteogenic potential, can improve surgical bed repair [18].

Thus, the objective of this study was to evaluate the capacity of mandibular bone marrow blood aspirate associated with biomaterials to stimulate bone tissue neoformation compared to the use of peripheral blood aspirate in patients with bone loss in the premaxillary region.

## 2. Methods

### 2.1. Study Design

The current study was designed as a double-blind, randomized, parallel arms controlled clinical trial study with the aim of evaluating the clinical, scintigraphic, and histological results of blood aspirate from mandibular bone marrow compared to peripheral blood in patients with atresia in the premaxillary region. The study was approved by the Research Ethics Committee (IRB—# 2,540,760) of the São Leopoldo Mandic Dental Research Institute and Center (Campinas, SP, Brazil). Prior to participation, all patients were individually informed about the nature and risks and benefits of the proposed study and signed an informed consent form.

### 2.2. Population Screening

A total of 16 patients (8 women and 8 men, average age of 50.56 ± 5.12 years old) were recruited in the present study. The inclusion criteria were edentulous patients in the premaxillary region, anterior alveolar ridge with a buccolingual thickness of 2-3 mm, need for rehabilitation with osseointegrated implants in the anterior maxillary region, and being aged between 40 and 60 years. The exclusion criteria were the history of neoplastic disease treated with radio or chemotherapy, pregnant or nursing patients, use of medication that changes the bone metabolism, smokers, and diabetic patients.

### 2.3. Randomization and Power Calculation

The sample size was based on previous studies [14, 19, 20], in which power calculation was performed using a statistical software, establishing that 16 patients should be included to reach 80% of power in detecting bone formation level differences between groups.

The patients were randomly divided with a 1 : 1 allocation ratio into the following two groups. Group 1 (G1), with only xenogenic bone blocks associated with peripheral blood and grafting in hemipremaxilla (*n* = 16). Group 2 (G2), with mandibular bone marrow blood associated with the same biomaterial (*n* = 16) on the contralateral side. As the study had a split mouth design, all patients received both treatments. The hemipremaxilla receiving each treatment was assigned randomly using the website Randomization.com (http://www.randomization.com), and the randomization was placed in an opaque envelope. All patients were enrolled and equally prepared for the surgical procedure at the same section by a single investigator (PJP). The procedures were only revealed to the surgeon immediately before the surgery.

### 2.4. Blinding

Study participants were blinded to the treatment received. Blinding of the investigator was applicable until the surgery section. Participants' identity and their corresponding study group were concealed by assigning an identification number to all laboratory specimens.

### 2.5. Surgical Procedure

Before the surgical procedure, each patient was prepared with intraoral mouthwash and perioral skin asepsis using 0.12% and 2% chlorhexidine, respectively. After placing sterile drapes, topical anesthesia with xylocaine was administered in the region to be surgically accessed, followed by infiltrative anesthesia and regional blockade with 1 : 100,000 mepivacaine and epinephrine association. Then, an incision was made in the crest of the alveolar ridge between the canine regions. A full-thickness flap was detached in the premaxilla with periosteum detachers, and the remaining bone structure was removed with number 2 spherical carbide drills in order to expose maxillary bone marrow and maximize graft irrigation. Subsequently, xenogenic blocks of bovine origin (Bonefill®, Bionnovation, Bauru, SP, Brazil) measuring 20 × 20 × 5 cm were sculpted with diamond disks and carbide drills to adapt them to the remaining bone base.

### 2.6. Mandibular Bone Marrow Blood Aspirate and Peripheral Blood Collection

In the same surgical procedure, the patient was anesthetized in the mandibular region, and 1 mL of blood was collected from the mandibular bone marrow region. Cone beam computed tomography was used to choose a wide and easily accessible bone marrow chamber. A 22 mm long steel drill with 2.3 mm diameter was used. This drill has a mobile metal ring in its composition that can be fixed to obtain a stop of safe depth, with a sharp active tip at the end to penetrate the cortical bone. A 10 mL hypodermic disposable syringe (BD) [21] adapted to a sterilizable metal cannula (ICE SP, Brazil) of 2 mm diameter previously heparinized was used to access the bone marrow chamber of the mandible and collect mandibular bone marrow blood.

For the peripheral blood sampling, a venipuncture was performed in the superficial veins of the anterior surface of the upper extremity. A tourniquet 3-4 inches above the selected puncture site was applied. The blood collection was accomplished using 22-gauge needles and 5 mL syringes without any additive (BD®, USA).

According to the groups evaluated, the sculpted blocks were filled with peripheral blood during surgery (G1) or dripped with mandibular bone marrow blood until the xenograft was completely wet (G2). After adaptation, the blocks were fixed to the bone defect with one or two titanium screws (Neodent, Curitiba, PR, Brazil) with 1.5 mm in diameter and 8.0–10.0 mm in length. After block fixation, a hydrogel membrane (Biocelltis, Florianópolis, SC, Brazil) was used to isolate the xenograft in both groups. Next, the wound was sutured with nylon 5 thread (Technofio, Goiânia, GO, Brazil).

### 2.7. Bone Scintigraphy

**Figure 1:**
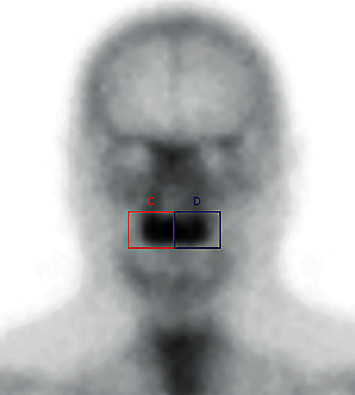
Representative image pixel measurement in the ROI.

On days 7 and 14 after grafting, bone scintigraphy was performed to comparatively analyze osteogenic cell activity in the grafted area in the groups [22–24] (Figure 1).

The scintigraphy device used was a single-headed Symbia E (Siemen, Germany). The test was performed through intravenous administration of the radiopharmaceutical methylene diphosphonate (MDP) labeled with technetium-99 m (99 mTc), with the mean dose administered being 26–30 mCi, calculated according to the patient's weight. MDP is a drug with tropism for organic bone matrix which concentrates in greater quantity with greater osteogenesis [25–27]. O 99mTc is a radioisotope that emits pure gamma radiation, with 140 Kev of energy and physical half-life of six hours.

The test was performed at the Nuclear Medicine Center of Santa Mônica Hospital (Erechim, RS, Brazil). The images obtained were evaluated considering the number of pixels in the region of interest (ROI).

The optical density of the ROI was evaluated using the e.soft syngo® analysis program (Siemens, USA).

### 2.8. Histological and Histomorphometric Analyses

The implants were installed 4 months after grafting. Tissue samples were collected using a trephine drill of 2.3 mm diameter in the axial direction of the implant installation site, up to a height of the remaining bone base. After sample collection, the implants (Bone Level Tapered, BLT, Straumann, Basileia, Switzerland) were installed sequentially in the following dimensions (3.3 mm diameter X 10.0 mm length).

The collected samples were stored in 10% buffered formalin (pH 7.2) and sent for histological evaluation at the Laboratory of Anatomical Pathology of Faculdade São Leopoldo Mandic (Campinas, SP, Brazil). Then, the samples were demineralized with 20% formic acid, embedded in histological paraffin, and stained with hematoxylin and eosin. Later, the slides were mounted with biological resin (Permount®, Fisher Scientific, NJ, USA).

Histomorphometry of the slides was performed from images captured in a computerized imaging system (AxioVision Rel 4.8, Carl Zeiss, Oberkochen, Germany) coupled to the Axioskop 2 plus optical light microscope (Carl Zeiss, Oberkochen, Germany).

**Figure 2:**
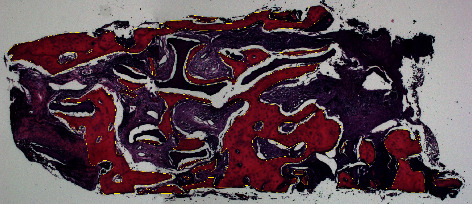
Histological image stained with hematoxylin and eosin showing the measured areas in the trephine, in yellow.

This analysis was performed by a single examiner blinded to the treatment performed (E.F.M). The entire trephined area was evaluated using the ImageJ image analysis program (National Institute of Health, Maryland, USA), and bone tissue areas were quantified as described by previous studies [14, 28, 29], measuring the areas occupied by newly formed bones in *µ*m^2^ (Figure 2).

### 2.9. Statistical Analysis

All the data were subjected to descriptive and exploratory analyses. Scintigraphic measurement data were subjected to the two-way analysis of variance post-Student–Newman–Keuls test. The data on newly formed bone tissue area and percentage did not meet the normality assumptions and were analyzed with the Wilcoxon paired test. A significance level of 5% was considered in all analyses.

## 3. Results

### 3.1. Bone Scintigraphy

The scintigraphic results of the groups evaluated are given in Table 1. There was a higher mean amount of pixels in G2 compared to G1 (*p* < 0.05) at both times evaluated.

### 3.2. Histological and Histomorphometric Analyses

**Figure 3:**
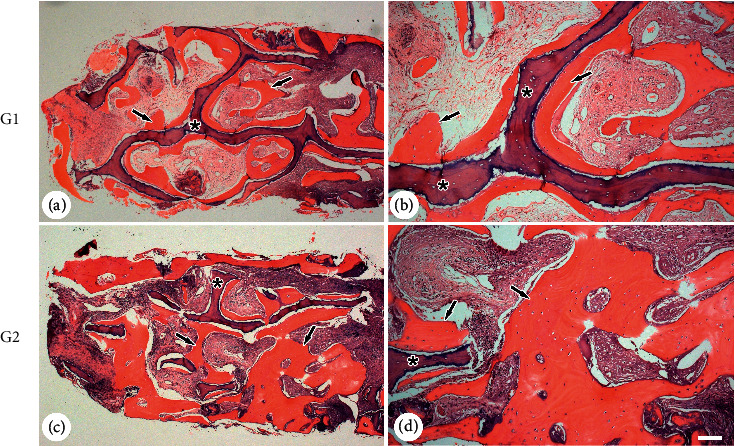
Histological images stained with hematoxylin and eosin from the trephined areas with different treatments. Detailed images show the presence of newly formed bone (⟶) and the presence of remaining biomaterials (*∗*). (a)-(b) Group 1 (G1), xenograft associated with peripheral blood. (c)-(d) Group 2 (G2), xenograft associated with mandibular bone marrow blood aspirate. Bars: (a) and (c) = 1,000 *µ*m; (b) and (d) = 100 *µ*m.

Histological and histomorphometric results are given in Table 2 and Figure 3. The percentage of bone tissue in the analyzed region was higher in G2 (47.09 (1.11)%) than in G1 (34.93 (1.60)%) (*p* < 0.05).

Representative histological images of the groups are shown in Figure 2. G2 presents a greater amount of newly formed bone tissue intermingled with particles of bone substitute compared to G1.

## 4. Discussion

The treatment of partial and total edentulous patients with dental implants has become a routine treatment modality in contemporary dental practice. However, tooth loss is often associated with bone loss, with insufficient bone dimensions for not only surgical installation of the implant but also for future prosthetic rehabilitation to place the dental implant in a prosthetically optimal position [30, 31].

In this context, bone grafting procedures play an important role in bone tissue recovery for implant placement. Among the options for bone grafting, autologous bone blocks are still considered the gold standard, especially in regions with critical bone defects. Due to its physical and biological constitution, autologous grafts present low association with immunological reactions, in addition to having the necessary pillars for balanced bone neoformation, such as osteoconduction, osteoinduction, and osteogenesis [32, 33]. However, studies seek the perfect biomaterial to replace this grafting format to minimize critical autogenous grafting problems, such as more than one surgical site, and limited amount of material [33].

Combined with the type of bone substitute, techniques that aim to strengthen the osteogenic potential of the biomaterial, especially by increasing its cellularity, have been a strategy to accelerate bone tissue formation, thus reducing the time to rehabilitate the patient [14, 19, 34, 35].

Thus, the objective of the present study was to evaluate the capacity of mandibular bone marrow blood aspirate associated with biomaterials to stimulate bone tissue neoformation compared to the use of peripheral blood aspirate in patients with bone loss in the premaxillary region. The clinical results showed that the association of bone marrow blood and the biomaterial increased osteogenic capacity and bone neoformation compared to peripheral blood.

Some studies show that bone marrow represents a promising source of autologous cells [5], improving its osteogenic potential [18, 36, 37] and providing a high concentration of growth factors [38–40], which are important to form a microenvironment to stimulate mineralized bone matrix formation.

Bone marrow blood is commonly collected from the iliac crest bone [41]. However, collection from this region requires a hospital environment, being a more invasive procedure for the patient with higher costs, risk of failure in the donor region, in addition to result in difficult preambulation [42]. Thus, in an attempt to obtain bone marrow blood in an easier way, in the clinical environment, at the same surgical time, and without risk to the patient, this study evaluated the osteogenic potential of the biomaterial soaked in bone marrow blood obtained from the mandibular region compared to peripheral blood present in the surgical bed.

It is important to note that the mandible was the bone of choice for obtaining bone marrow blood, as research has shown the greater osteogenic potential of cells from this region compared to those generally obtained from long bones [17, 43–45], in addition to greater periosteal cell viability [46, 47]. Viable vascularized and well-perfused bone graft is a prerequisite for graft survival; however, there is little evidence that graft viability is related to a successful clinical outcome [21, 48, 49]. Radioisotope bone scintigraphy has been proven to be an effective method to evaluate vascular patency and bone viability, especially in orthopedic surgeries [50, 51]. In the present study, scintigraphic results showed a higher mean number of pixels with the association of bone substitute with mandibular bone marrow blood aspirate compared to peripheral blood (*p* < 0.05), which reinforces greater vascularization and greater osteogenic cell activity in the initial phase of grafting [43, 52] due to greater radiopharmaceutical fixation and the consequent number of pixels in G2 compared to G1.

In addition to the clinical findings, the histomorphometric results obtained from biopsy analysis confirmed a greater amount of bone tissue in the group using mandibular bone marrow blood, showing a 34.93% higher bone neoformation compared to the association of bone substitute with peripheral blood. These findings reinforce that mandibular bone marrow blood has unique properties, which differentiates it due to its osteogenic potential compared to peripheral blood [39, 45, 53], which is conventionally used in surgery. In addition to its reparative property, it is easier to use compared to other spinal cord blood donor regions, reducing comorbidities [1] and being an interesting alternative for grafting procedures.

## 5. Conclusions

Appositional bone reconstruction using blocks of bone substitute associated with mandibular bone marrow blood induced greater osteogenic activity and bone neoformation than the association with peripheral blood, enabling more conservative surgical procedures.

## Figures and Tables

**Table 1:** Mean scintigraphic analysis of the groups evaluated at the different analysis times, in pixels.

	G1	G2
7 days	28239.00 (43.26)^B, b^	30833.00 (106.71)^A, b^
14 days	29483.67 (92.96)^B, a^	31781.33 (127.93)^A, a^

Different capital letters represent differences between groups at each time (horizontal). Different lowercase letters represent differences between times in each group (vertical). Significance level 5%. Group 1 (G1), xenograft associated with peripheral blood; Group 2 (G2), xenograft associated with mandibular bone marrow blood aspirate.

**Table 2:** Histomorphometric analysis as a function of treatment.

Variable	Treatment				*P* value
	G1		G2		
	Mean (standard deviation)	Median (minimum and maximum value)	Mean (standard deviation)	Median (minimum and maximum value)	
Bone tissue area (*µ*m^2^)	977.13 (235.33)	884.50 (764.00; 1435.00)	1838.38 (1004.60)	1286.00 (970.00; 3234.00)	0.0499
% of bone tissue	34.93 (1.60)^a^	34.88 (32.91; 37.22)	47.09 (1.11)^b^	46.86 (45.74; 49.21)	0.0117

Different letters represent differences between groups. Significance level 5%. Group 1 (G1), xenograft associated with peripheral blood; Group 2 (G2), xenograft associated with mandibular bone marrow blood aspirate.

## Data Availability

The data used to support the findings of this study are available from the corresponding author upon request.

## Supplementary Materials

Supplementary Materials Flow diagram for patient's eligibility and inclusion in the study (*s*).
