# Supplementary material for: Clinical, Histological, and Scintigraphic Comparative Study of the Use of Mandibular Bone Marrow and Peripheral Blood in Bone Neoformation
Source: Int J Dent. 2021 Dec 31;2021:4867574. doi: 10.1155/2021/4867574 (PMC8741402; doi:10.1155/2021/4867574)
Supplement: Supplementary Materials — Flow diagram for patient's eligibility and inclusion in the study (s). [file 4867574.f1.doc]

**Supplementary file.** Flow diagram for patient's eligibility and inclusion in the study.

**Allocation**

**Analysis**

**Follow-Up**

**Enrollment**

Assessed for eligibility (n= 25)

Excluded (n= 9)

♦  Not meeting inclusion criteria (n= 9)

Analysed (n= 16)
♦ Excluded from analysis (n= 0)

Discontinued intervention (n= 0)

Randomized (n= 16)

Allocated to intervention (n= 16)

♦ Received allocated intervention (n= 16)

Allocated to intervention (n= 16)

♦ Received allocated intervention (n= 16)

Discontinued intervention (n= 0)

Analysed (n= 16)
♦ Excluded from analysis (n= 0)
